# Expression and Clinical Significance of Plasma miR-223 in Patients with Diabetic Nephropathy

**DOI:** 10.1155/2023/9663320

**Published:** 2023-12-27

**Authors:** Xingrong Guo, Meiying Huang, Dawei Yang, Zuojie Luo

**Affiliations:** ^1^Department of Endocrinology, The First Affiliated Hospital of Guangxi Medical University, Nanning, Guangxi, China; ^2^Department of Endocrinology, The Affiliated Hospital of Youjiang Medical University for Nationalities, Baise, Guangxi, China; ^3^Department of Nephrology, The Affiliated Hospital of Youjiang Medical University for Nationalities, Baise, Guangxi, China; ^4^Department of Geriatric Medicine, The Affiliated Hospital of Youjiang Medical University for Nationalities, Baise, Guangxi, China

## Abstract

**Background:**

MicroRNA-223 (miR-223) is associated with diabetes and kidney diseases and serves as a novel marker for diagnosing diabetic kidney disease (DKD). This study was conducted to investigate the plasma expression of miR-223 and its clinical significance in type 2 diabetes (T2DM) and diabetic nephropathy (DN) patients.

**Methods:**

In this research, 20 patients with T2DM and DN, 19 patients with T2DM, and 17 healthy volunteers were finally enrolled. miR-223 expression was detected by quantitative real-time PCR (qPCR), and the diagnostic value of miR-223 in DN was further analyzed.

**Results:**

miR-223 was downregulated in the DN group compared to that in the T2DM group (*P*=0.031) and the control group (*P* < 0.001). Pearson's correlation analysis showed a negative correlation of miR-223 levels with an albumin-creatinine ratio (ACR) (*r* = −0.481; *P*=0.044), urine *β*2-microglobulin (*β*2-MG) (*r* = −0.494; *P*=0.037), urine *α*1-microglobulin (*α*1-MG) (*r* = −0.537; *P*=0.022), creatinine (Cr) (*r* = −0.664; *P* < 0.01), cystatin C (Cyc-C) (*r* = −0.553; *P*=0.017), and glycosylated hemoglobin (HbA1c) (*r* = −0.761; *P* < 0.01). The findings of a binary regression analysis indicated that miR-223, ACR, Cr, and *α*1-MG were the risk factors for DN (OR: 2.019, 1.166, 1.031, and 1.031; all *P* < 0.05). Furthermore, miR-223 had a favorable diagnostic value for DN (AUC: 0.752; sensitivity: 0.722; specificity: 0.842) (2.5 was utilized as the diagnostic cutoff point).

**Conclusion:**

miR-223 was lowly expressed in DN patients, and the evaluation of miR-223 may be a good approach for diagnosing DN.

## 1. Introduction

The prevalence of diabetes mellitus (DM) in China is increasing [1]. Many articles have revealed that about 40% of DM patients are complicated with diabetic kidney disease (DKD) [2, 3], and these patients will suffer from end-stage renal disease (ESRD) if untreated. During 2000–2015, the annual prevalence of ESRD among DM patients worldwide elevated from 375 million to 1,016 million [4]. DKD, as a severe complication of DM, is also a primary public health burden [5] and a chief cause of ESRD worldwide [6]. Based on this, the recognition of hallmarks predicting DKD risk has the greatest relevance for DM patients.

Lately, there has been an increasing interest in the capability of small noncoding RNAs (20–22 bases), known as microRNAs (miRNAs). miRNAs are remarkably stable in blood [7] together with other body fluids [8]. Given the stability of miRNAs and the minimal invasive property for the sampling of blood and body fluids, they may be helpful as targets for diagnosis and/or prognosis. miRNAs mainly modulate messenger RNA (mRNA) function via specific binding to target mRNAs, thereby causing mRNA degradation and protein translation suppression by epigenetic events [9]. In a large number of newly identified miRNAs, microRNA-223 (miR-223) is abnormally expressed in patients with acute cerebral infarction, ankylosing spondylitis, gastric malignancy, periodontitis, lipid metabolism, and obesity [10–15]. Current studies uncover that miR-223-3p might be linked to glucose metabolism, type 2 diabetes mellitus (T2DM), and gestational diabetes mellitus (GDM) [16–20]. miR-223 may serve as a hallmark and potential therapeutic target for diabetes, cardiovascular disease, and penile cancer [21–23]. miR-223 expression was reduced in T2DM subjects in contrast to impaired glucose tolerance (IGT), impaired fasting glucose (IFG), or control subjects [24]. Notably, many miRNAs have been revealed to directly link to several T2DM-related clinical traits, such as plasma glucose, insulin, A1c, and homeostatic model assessment of insulin resistance (HOMA-IR), which are also the prediction of developing T2DM [25].

The study of Ulbing et al. [26] signified that miR-223-3p expression was decreased in renal transplantation patients with chronic kidney disease (CKD) stages 4 and 5 in contrast to healthy controls. However, the study enrolled ESRD patients from various causes, and the sample size of DKD patients was small. Meanwhile, patients with ESRD have multiple complications, and whether these complications affect the results needs further study. The purpose of this paper was to excavate the capability of miR-223 in DKD and its underlying pathogenesis through the recognition of reliable and novel biomarkers.

## 2. Methods

### 2.1. Study Design and Participants

Collectively, 20 patients with T2DM and DN (DN group), 19 patients with T2DM (T2DM group), and 17 healthy volunteers (control group) who pursued medical advice in the Affiliated Hospital of Youjiang Medical University for Nationalities between January 2021 and September 2021 were included in this cross-sectional study. We defined DKD patients as prevalent albuminuria (with a urinary albumin-to-creatinine ratio more than or equal to 30.0 mg/g) and with retinopathy following the Diabetes Management in Chronic Kidney Disease: Synopsis of the 2020 KDIGO Clinical Practice Guideline [27] and the China T2DM prevention and treatment guidelines 2021 [28].

Patients with urinary calculi, cysts, peripheral vascular disease-induced nephropathy, nephritis caused by other diseases, and other serious diseases influencing glycemia or albuminuria (e.g., acute infection, pregnancy, significant organ failure, malignancy, and medications) and patients with ESRD were excluded. Healthy volunteers were those without DM and evidence of any kidney disease. DM was ruled out using the glucose tolerance test.

The Ethics Committee of the Affiliated Hospital of Youjiang Medical University for Nationalities approved the protocol of this study (YYFY-LL-2022-006). Written informed consent was acquired for each participant.

### 2.2. Data Collection

Anthropometrical and biochemical parameter measurements: Basic and clinical traits including sex, age, and disease duration, and laboratory data, such as fasting blood glucose (FBG), glycated hemoglobin (HbA1c), creatinine (Cr), estimated glomerular filtration rate (eGFR) (computed using the CKD-EPI equation) [29], total glycerides (TGs), low-density lipoprotein cholesterol (LDL-C), cystatin C (Cyc-C), urine *β*2-microglobulin (*β*2-MG), urine *α*1-microglobulin (*α*1-MG), and a urinary albumin-creatinine ratio (ACR), were collected. The calculation of the body mass index (BMI) was achieved by body weight/body height^2^ (kg/m^2^). The Cr, HbA1c, and blood lipid concentrations were appraised by using Mindray Automatic Biochemical Analyzer BS-2000M (Guangzhou, China). A magnetic particle chemiluminescence instrument (Autobio) was implemented for insulin measurement. ACR and *β*2-MG were estimated by Specific Proteins Analyzer BA400 (BioSystems, Spain). SI units were applied accordingly.

miR-223 expression via a real-time PCR analysis: Peripheral blood samples of all participants were collected. The venous blood (5 ml) was drawn from every patient using anticoagulant vacutainer tubes and centrifuged for 15 minutes in a 4° cryogenic centrifuge at 2500 rpm, after which the plasma was separated, and the supernatant was absorbed. TRIzol™ Ls reagent (Invitrogen, USA) was employed for isolating total plasma RNA. Subsequently, genomic DNA was removed, and hiscript ®IIQRT Supermix for qPCR (+gDNA wiper) kit (Vazyme, Nanjing, China) was utilized for reverse transcription. The primers for reverse transcription were procured from Optimaceae. All the aforesaid procedures were implemented under the kit's requirements.

Quantitative real-time PCR: SYBR Green Master Mix (Vazyme, Nanjing, China) was implemented for PCR analysis. The PCR reaction system was as follows: 10 min predenaturation at 95°C, 1 cycle; 15 s denaturation at 95°C, 60 s annealing extension at 60°C, 40 cycles; melting curve acquisition: 95°C 50 s, 60°C, 60 s, 95°C, 15 s, 1 cycle. The amplification curve and the dissolution curve were analyzed. The average value of three times of data for every sample was utilized for analysis. The miR-223 level was estimated by the 2^−ΔΔCt^ method, with GADPH as a loading control.  Table 1 displays the primer sequences.

### 2.3. Statistical Methods

SPSS software, version 25.0 (IBM, Armonk, NY, USA), was employed for data analysis. Continuous variables were expressed as the mean ± standard deviation (SD). The relevance of miR-223 expression with each clinicopathological element was analyzed with the chi-square test. The correlation of miR-223 levels with glomerular filtration rate and proteinuria, along with other clinicopathological traits, was processed with Pearson's correlation coefficient. The parameters with differences were enrolled in the binary regression analysis for excavating the risk indicators of DN in T2DM patients. Besides, the diagnostic cutoff point, sensitivity, and specificity of each index were analyzed for DN identification by using the ROC curve. Statistical significance was interpreted as *P* < 0.05.

## 3. Results

### 3.1. Clinical Data in Three Groups

A total of 56 participants were recruited, comprising 19 T2DM patients (9 men and 10 women; average age: 54.85 ± 14.24 years), 20 DN patients (11 men and 9 women; average age: 56.30 ± 12.67 years), and 17 nondiabetic healthy volunteers (8 men and 9 women; average age: 49.57 ± 12.54 years). No differences were witnessed in disease duration, BMI, TG, LDL-C, urine *β*2-MG, gender, and age in all three groups (all *P* > 0.05), while a marked difference was observed in Cr, FBG, ACR, eGFR, Cyc-C, *α*1-MG, and A1c (all *P* < 0.05) (Table 2).

### 3.2. miR-223 Differential Expression in Three Groups

**Figure 1:**
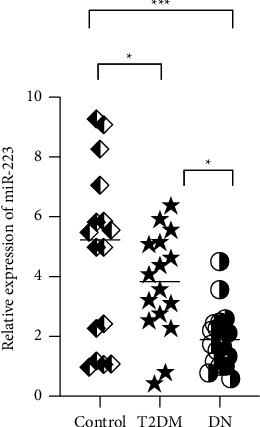
Expression of circulation miR-223 in DN patients, T2DM patients, and healthy controls. miR-223 was downregulated in the DN group compared to that in the T2DM group and the control group.

A distinct difference in miR-223 expression was observed in the three groups (*P* < 0.01). There was a lower miR-223 level in T2DM and DN patients than in healthy controls (*P*=0.031, *P* < 0.001, respectively) (Figure 1). Subsequent analysis revealed that a lower miR-223 level was observed in DN patients than in T2DM patients (*P*=0.020) (Figure 1).

### 3.3. miR-223 Level in Injured Renal Tubules

**Figure 2:**
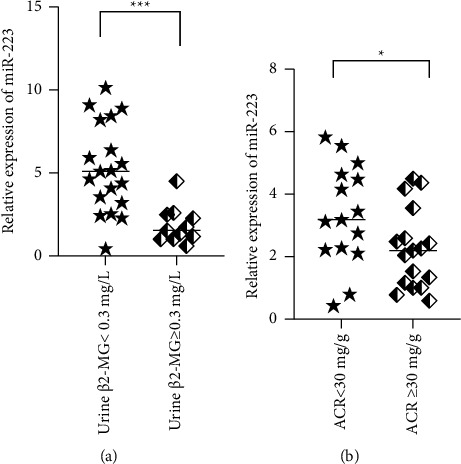
(a) Association of miR-223 levels with the degree of urine *β*2-MG. Urine *β*2-MG readout <0.3 mg/L was normal, while ≥0.3 mg/L abnormal, an elevated serum miR-223 level in T2DM patients with abnormal urine *β*2-MG compared to those with normal urine *β*2-MG. (b) Association of miR-223 levels with the degree of ACR. ACR readout <30 mg/g was defined as normal, while ≥30 mg/g abnormal. T2DM patients with abnormal ACR exhibited an elevated serum miR-223 level compared to those with normal ACR.

Based on the findings of the miR-223 level in renal tubule urine *β*2-MG, we observed an elevated serum miR-223 level in T2DM patients with abnormal urine *β*2-MG compared to those with normal urine *β*2-MG (*t* = 4.049, *P* < 0.001; Figure 2(a)).

The outcomes of the miR-223 level in ACR disclosed that T2DM patients with abnormal ACR exhibited an elevated serum miR-223 level compared to those with normal ACR (*t* = 2.149, *P*=0.040; Figure 2(b)).

### 3.4. Relevance of miR-223 Levels in T2DM Patients with *α*1-MG, Cr, Cyc-C, ACR, *β*2-MG, and HbA1c

**Figure 3:**
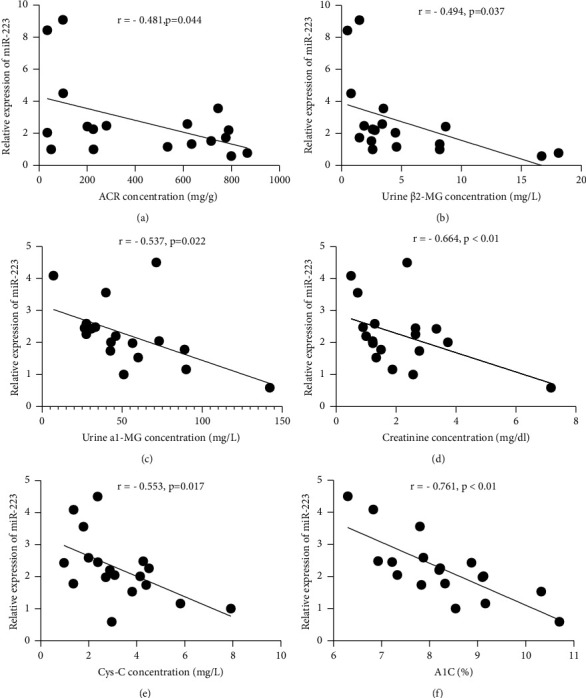
Correlation analysis of miR-223 expression with the concentration of ACR, urine *β*-MG, urine *α*1-MG, creatinine, Cys-C, and HbA1c. (a) miR-223 level was negatively correlated with ACR (*r* = − 0.481). (b) miR-223 level was negatively correlated with urine *β*2-MG (*r* = − 0.494). (c) miR-223 level was negatively correlated with urine *α*1-MG (*r* = − 0.537). (d) miR-223 level was negatively correlated with Cr (*r* = − 0.664). (e) miR-223 level was negatively correlated with Cys-C (*r* = − 0.553). (f) miR-223 level was negatively correlated with A1c (*r* = − 0.761).

The relevance of miR-223 levels in T2DM patients with *α*1-MG, Cr, Cyc-C, ACR, *β*2-MG, and HbA1c was processed with Pearson's linear correlation coefficient. It was signified that the miR-223 level harbored a negative relevance to *α*1-MG, Cr, Cyc-C, ACR, *β*2-MG, and HbA1c (Figures 3(a)–3(f)).

### 3.5. Clinical Value of miR-223 Levels in Peripheral Blood for Diagnosing DN

**Figure 4:**
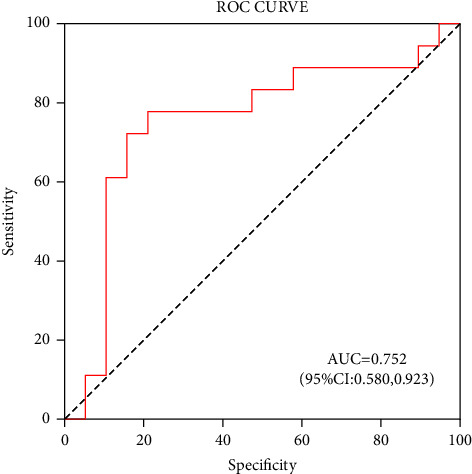
ROC curve analysis of miR-223 in identifying DN in patients with type 2 diabetes mellitus. miR-223 had a favorable diagnostic power for DN (AUC: 0.752 (95% CI: 0.580–0.923)).

Binary regression analysis unveiled that miR-223(2^−ΔΔCt^), ACR, Cr, and *α*1-MG were determined to be the risk indicators for DN (OR: 2.019, 1.166, 1.031, and 1.031; all *P* < 0.05). Furthermore, miR-223 had a favorable diagnostic power for DN (AUC: 0.752 (95% CI: 0.580–0.923); sensitivity: 0.722; specificity: 0.842). The best diagnostic cutoff point miR-223 level was found at <2.5 (Figure 4).

## 4. Discussion

T2DM remains highly common in China [1]. Kidney injury, as a frequent complication of T2DM, influences more than a third of the 100 million diabetic people in China [1]. DKD itself is expensive to treat, and it is also linked to enhanced risks of cardiovascular events and fatality [30]. DKD poses enormous human, economic, and societal burdens, and more people will suffer from DKD due to the elevated number of T2DM patients in China [31, 32]. Although the molecular mechanism of DKD pathogenesis is not well understood, accumulating evidence has uncovered the pivotal role of genetic regulation. The essential roles of miRNAs in multiple diseases have been unveiled in many publications [33]. As previously described, miR-223 dysregulation is observed in CKD progression, which might be a diagnostic target. Currently, there exist no effective methods to prevent DKD patients from developing ESRD. There is an urgent need to discover novel biomarkers to facilitate the early diagnosis and effective treatment in DKD patients. It is then possible to intervene ESRD development in DKD patients.

In this paper, notable downregulation of miR-223 was witnessed in T2DM and DN patients in contrast to healthy controls, which was in agreement with Ulbing et al.' study [26]. DKD is asymptomatic in the initial stage and deteriorated slowly, leading to kidney enlargement and elevated GFR [34]. The elevation in GFR is accompanied by changes in the filtration membrane charges and the presence of urine microalbumin (mAlb) along with disease progression. However, GFR is still in the normal range, without any relevant clinical symptoms. The only abnormality is found in urine mAlb. To date, mAlb is a vital parameter for the diagnosis of early DKD. ACR is usually utilized for correcting the fluctuations of random urinary mAlb levels in clinical practice. In our work, *α*1-MG, Cr, Cyc-C, ACR, and *β*2-MG were chosen for evaluating the kidney function conditions of DN and T2DM patients. We observed an elevated serum miR-223 level in T2DM patients with abnormal *β*2-MG compared to those with normal urine *β*2-MG. Meanwhile, T2DM patients with abnormal ACR exhibited an elevated serum miR-223 level compared to those with normal ACR. Moreover, it was signified that the miR-223 level harbored a negative relevance to *α*1-MG, Cr, Cyc-C, ACR, *β*2-MG, and HbA1c, revealing the involvement of miR-223 in kidney impairment. An acute and chronic proinflammatory state exists in patients with chronic kidney disease (CKD), contributing substantially to morbidity and mortality. Interleukin-6 (IL-6) is a key inflammatory factor in kidney disease and is also recognized as a diagnostic marker and therapeutic target [35, 36]. IL6ST is a signaling transducer of the IL-6 cytokine family. The activation of the IL6 receptor leads to the formation of the IL6ST dimerization initiation signaling platform, ultimately activating the JAK-MAPK and JAK-STAT3 signaling pathways [37]. miR-223-3p directly binds to IL6ST mRNA 30UTR to downregulate the mRNA and protein expressions. This may inhibit the signaling process mediated by IL-6, including the phosphorylation of STAT3, which is consistent with the view that targeting IL-6-dependent signaling can alleviate the harmful effects of kidney disease [38].

Our work also disclosed the negative relevance of the miR-223 level to Cyc-C. The stages of CKD are defined following the Improving Global Outcomes (KDIGO) via eGFR, barrier function (proteinuria), and Cr [39], which have been proven to be useful. Nevertheless, there are some drawbacks; for example, healthy kidney aging is incorrectly classified as CKD. Cyc-C is an ideal homologous marker reflecting the change in glomerular filtration rate, which is not affected by gender, diet, and other factors [40]. The combination of Cys-C, ACR, and miR-223 may be used as a good prediction model for early diagnosis and treatment of DN.

Binary regression analysis unveiled that miR-223 (2^−ΔΔCt^), ACR, Cr, and *α*1-MG were determined to be the risk indicators for DN (OR: 2.019, 1.166, 1.031, and 1.031; all *P* < 0.05). Furthermore, miR-223 had a favorable diagnostic power for DN (AUC: 0.752 (95% CI: 0.580–0.923); sensitivity: 0.722; specificity: 0.842). It can be seen that the surface miR-223 expression in peripheral blood has a high diagnostic value in differentiating DN patients and healthy people and has high sensitivity and specificity for diagnosing DN.

Initially, this work signified a low miR-223 level in the circulating of DN patients and miR-223 exhibited relevance to ACR, Cr, Cyc-C, urine *β*2-MG, and urine *α*1-MG. This research would further profit from the theories of DKD pathogenesis and supply novel hallmarks for DKD gene-targeted therapy. Meanwhile, our discoveries offer references for pursuing biomarkers for DKD diagnosis and prognosis and supply a novel regimen for clinically refractory DKD. Nonetheless, DKD is caused by multiple factors, and the definite pathogenesis, diagnosis, and outcome evaluation should be further deciphered.

This work has certain limitations. At first, it was a single-center study. Second, multivariable analysis was not performed in this study. Therefore, the results need further experimental verification. Third, the relationship between miR-223 and other inflammatory nephritis needs further study. However, this study provided clues for early diagnosis of DKD by correlation analysis between plasma miR-223 and ACR, *β*2-MG, *α*1-MG, Cr, and Cyc-C.

In conclusion, this study demonstrated that miR-223 had low expression in the circulation of DN patients and harbored a negative relevance to *α*1-MG, Cr, ACR, *β*2-MG, and Cyc-C.

## Figures and Tables

**Table 1:** Primer sequence.

Genes	Primer	Sequence (5′-3′)
hsa-mir-223	Forward	GTCGTATCCAGTGCAGGGTCCGAGGTATTCGCACTGGATACGACTGGGGTAT
	F primer	TGCGCTGTCAGTTTGTCAAATA

GAPDH	Forward	TCAAGAAGGTGGTGAAGCAGG
	Reverse	TCAAAGGTGGAGGAGTGGGT

miR-223, microRNAs223; GAPDH, glyceraldehyde-3-phosphate dehydrogenase.

**Table 2:** Comparisons of basic clinical data in three groups.

Characteristic	Healthy control group (*n* = 17)	T2DM group (*n* = 19)	DN group (*n* = 20)	*P* value
Male, *n* (%)	8 (47.1%)	9 (47.4%)	11 (55.0%)	0.949
Age (years)	49.6 ± 12.5	54.9 ± 14.2	56.3 ± 12.7	0.300
Disease duration (years)	0	5.2 ± 1.5	6.8 ± 1.3	0.086
BMI (kg/m^2^)	24.0 ± 5.9	25.3 ± 4.5	24.4 ± 4.3	0.720
FBG (mmol/L)	5.2 ± 0.7	16.1 ± 8.1	12.9 ± 6.2	<0.001
A1c (%)	5.3 ± 0.48	10.8 ± 3.11	10.4 ± 2.81	<0.001
uACR (mg/g)	0.9 ± 0.8	2.1 ± 2.8	294.1 ± 271.2	<0.001
Cr (umol/L)	46.6 ± 6.8	62.1 ± 13.9	151.7 ± 120.4	<0.001
eGFR (ml/min)	111 ± 48.1	95.9 ± 35.3	74.4 ± 38.6	0.020
TG (mmol/L)	1.5 ± 0.5	1.9 ± 0.6	2.7 ± 3.1	0.180
LDL-C (mmol/L)	2.99 ± 0.72	3.49 ± 0.84	3.46 ± 1.60	0.353
Urine *β*2-MG (mg/L)	2.0 ± 4.6	6.6 ± 13.7	7.0 ± 12.1	0.390
Urine *α*1-MG (mg/L)	7.1 ± 9.4	19.2 ± 18.9	36.6 ± 34.5	0.003
Cys-C (mg/L)	0.6 ± 0.1	0.9 ± 0.1	3.1 ± 0.9	<0.001

BMI, body mass index; FBG, fasting blood glucose; A1c, glycated hemoglobin; uACR, urinary albumin-creatinine ratio; Cr, creatinine; eGFR, estimated glomerular filtration rate; TG, total glycerides; LDL-C, low-density lipid; *α*1-MG, *α*1-microglobulin; *β*2-MG, *β*2-microglobulin; Cyc-C, cystatin C.

## Data Availability

All data generated or analyzed during this study are included in this published article.
